# A New Management Technique for Symptomatic Haematomas Following Therapeutic Vacuum-Assisted Biopsy

**DOI:** 10.3390/jcm8091493

**Published:** 2019-09-19

**Authors:** Florentina Guzmán-Aroca, Juan de Dios Berná-Serna, Ana Azahara García-Ortega, Dolores Hernández-Gómez, Juan de Dios Berná-Mestre

**Affiliations:** 1 Department of Radiology, Virgen de la Arrixaca University Clinical Hospital, Ctra. Madrid-Cartagena, 30120 El Palmar (Murcia), Spain; jdberna@gmail.com (J.d.D.B.-S.); anaazaharagarcia@gmail.com (A.A.G.-O.); lhgomez00@hotmail.com (D.H.-G.); mesjubermu@hotmail.com (J.d.D.B.-M.); 2 Instituto Murciano de Investigación Biosanitaria Virgen de la Arrixaca (IMIB-Arrixaca), 30100 Murcia, Spain

**Keywords:** haematoma, breast, benign lesions, vacuum-assisted biopsy, ultrasound

## Abstract

The aim of this study was to investigate the efficacy of the vacuum-assisted biopsy (VAB) system in evacuating symptomatic haematomas after VAB excision of benign breast lesions. We retrospectively analysed the data of eight patients with symptomatic and large haematomas who were treated with VAB evacuation between 10 and 14 days after VAB excision. Only one case underwent the procedure 24 h after VAB excision, due to the patient reporting intense pain, which was relieved after application of the technique, even though it had to be done twice. This new clinical application of the VAB system for evacuating symptomatic breast haematomas was successful in all the cases in the present study. No technique-related complications were observed. Conclusions: To the best of our knowledge, this is the first study to reveal that VAB evacuation of symptomatic haematomas is safe, effective, quick and well-tolerated by patients.

## 1. Introduction

Benign or probably benign breast lesions are detected more and more often by women themselves as a casual finding and also upon ultrasound (US) examination due to the increase in US studies in young females and in the evaluation of lesions detected by breast cancer screening programmes [1,2]. Benign breast lesions cause a great deal of uncertainty and anxiety among women, who often demand surgical excision of the lesion. The vacuum-assisted biopsy (VAB) technique is used as a cost-effective alternative to surgery for excising benign breast lesions and offers diagnosis and treatment in the same intervention [1,3,4,5,6]. The indications for VAB with a therapeutic aim include (1) patients with a low probability of regular follow-up, (2) those planning a pregnancy, (3) extremely restless patients, (4) patients with a lesion increasing in size during follow-up, (5) patients who would prefer removal but do not want to undergo surgical excision for fear of a bad cosmetic result, (6) patients who have subjective symptoms or pain with Breast Imaging Reporting and Data System (BI-RADS) categories 3 and 4a lesions, (7) patients with family history of breast cancer and those carrying the BRCA1 or BRCA2 gene, and (8) dialysis patients awaiting a kidney transplant [1,7,8].

The most common complication after VAB in the treatment of benign breast lesions is a haematoma [5,8]. The management of breast haematomas is usually conservative, although percutaneous drainage is recommended for symptomatic and voluminous haematomas. However, US-guided percutaneous drainage with a catheter often leads to failure in our experience, especially in the first few weeks. We consider the VAB system to be an efficient technique for evacuating breast haematomas that are difficult to drain using conventional methods.

To our knowledge, this is the first study to describe VAB as a technique for evacuating symptomatic haematomas following VAB excision of benign breast lesions. The aim of this study was to investigate the efficacy of this technique in the management of these haematomas.

## 2. Material and Methods

### 2.1. Patients

A retrospective study was conducted between January 2014 and January 2019 on 1246 patients undergoing US-guided percutaneous VAB excision of BI-RADS 3 (*n =* 957) and BI-RADS 4a (*n =* 289) breast lesions, categorized using the American College of Radiology Breast Imaging Reporting and Data System [9]. Included in this study were patients aged over 18 years who had haematomas secondary to VAB measuring >25 mm in maximum diameter and who reported moderate or intense pain (between 4 and 7) on the visual analogue scale (VAS) from 0 to 10, where 0 meant no pain and 10 meant the worst possible pain. We excluded patients with haematomas of ≤25 mm or >25 mm and with a VAS pain of <4. The patients in the present study required analgesic (paracetamol). The study was conducted according to the guidelines of the Helsinki Declaration and was approved by the Institutional Review Board. Written informed consent was obtained from all the patients. 

### 2.2. Description of Procedure

**Figure 1:**
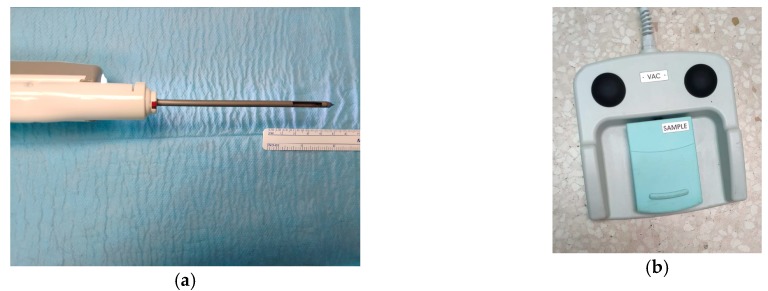

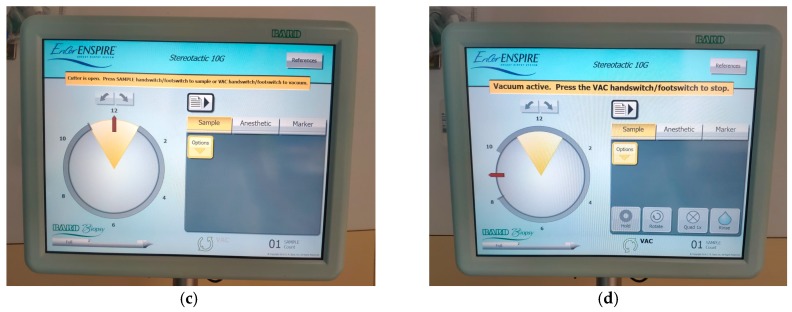
Example of a 10G needle with 20 mm aperture (**a**) and a pedal that includes vacuum (black) and cut (sample) buttons (**b**). The cutting needle opened at the point labeled 12 (**c**) and the direction for aspiration was clockwise (**d**).

The procedure for evacuating haematomas with the VAB system was performed 10–14 days after VAB excision of the breast lesion. Only one patient reported intense pain after excision of the lesion, and evacuation of the haematoma was performed at 24 h to reduce the pain. US examinations were performed by two radiologists with 6 and 29 years’ breast imaging experience, respectively, using an Acuson S2000 ultrasound system (Siemens, Erlangen, Germany) equipped with an 18L6HD transducer or Philips EPIQ 7 with a 18–5L MHz (Philips Healthcare, Bothell, WA, USA). Rigorous aseptic measures were applied. Local anaesthetic (2% mepivacaine) was injected into the probe puncture site (2 mL). A 14G Abbocath was then inserted into the haematoma cavity, but the method failed in all cases. This catheter was used to inject anaesthetic into the cavity (2–3 mL) and subsequently removed. The haematoma was then evacuated using a Mammotome® Revolve™ or EnCor® device with 10G needles, using first of all the empty-needle function and then the tissue-biopsy option (Figure 1), which is the sequence used initially, as it is impossible to evacuate the haematoma with just the use of the empty-needle mode; the empty-needle option is continued until the haematoma is resolved, although it is occasionally necessary to use the tissue-biopsy mode more than once. In all cases, the cutting needle opened at the point labeled 12 and the direction for aspiration was clockwise. It is not possible to standardize the empty-needle function or the number of cuts of tissue-biopsy option because each hematoma has a different size; it is normal to need one or two cuts (samples) with the rest being aspirated (empty-needle function). The haematoma site and probe trajectory were then compressed for 5–10 min. Finally, the puncture site was covered with a dressing and an instant cold pack was applied. Patients were scheduled for follow-up at 24 h, 1 or 2 weeks, and 6 months after the procedure. Success of the procedure was defined when the volume of the haematoma was reduced by >50% and the pain reported by the patients on the VAS scale was <4.

### 2.3. Data Collection

Demographic data were collected, such as participant age, BI-RADS category of the excised lesions, calibre of the needle used, maximum diameter and volume of the lesion, number of cylinders removed and histological findings of the 8 lesions in the present study. A haematoma was defined as a fluid collection of 20 mm in maximum diameter at the removal sites on the US examinations performed at 24 h [8]. The time between VAB excision of the lesion and VAB evacuation of the haematoma was recorded. Also recorded were the US appearance of the haematoma and the maximum diameter and volume using the formula V = 4/3 × π × (A/2 × B/2 × C/2) (A: longitudinal, B: transverse, C: antero-posterior) [4]. The time of the VAB procedure to evacuate the haematoma was also determined. At the end of the US procedure, pain intensity was registered using a VAS. These parameters were recorded again in the US follow-up 24 h after evacuation of the haematoma.

### 2.4. Statistical Analysis

Statistical analyses were performed using SPSS 24.0 (IBM SPSS Statistics, New York, NY, USA). A descriptive statistical analysis of each variable was conducted to give the frequency of distribution. The usual parameters were also calculated for the quantitative variables: mean ± standard deviation, maximum and minimum. Spearman’s rho was used to correlate quantitative variables, and the Mann–Whitney U test to compare dichotomous variables with quantitative variables. A *p*-value of <0.05 was considered statistically significant. 

## 3. Results

**Figure 2:**
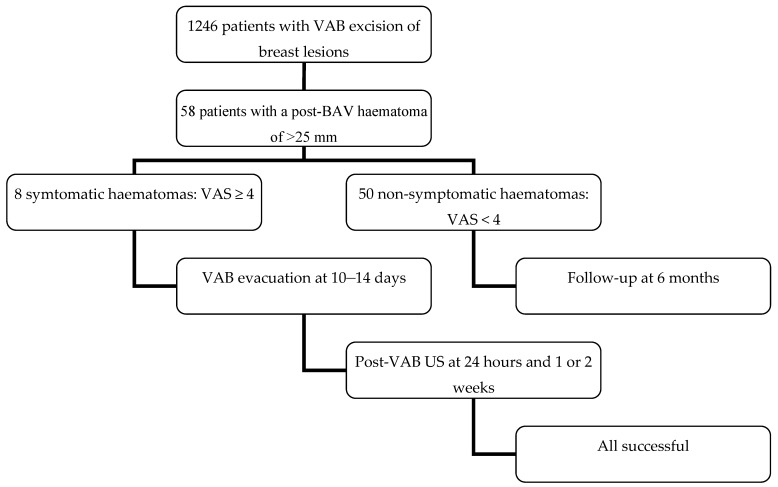
Flow diagram of patient inclusion in this study. VAB: vacuum-assisted biopsy; VAS: visual analogue scale; US: ultrasound.

The mean age ± SD of the 1246 patients undergoing VAB excision of the lesions was 47 ± 14.4 years (range: 18–67 years). Fifty-eight cases (4.6%) had a haematoma at the site of the lesion measuring >25 mm. Of the 58 cases, 50 reported a pain level of <5, and in eight cases, the pain was ≥5 (Figure 2). Table 1 shows the characteristics of the eight patients included in this study. The mean maximum diameter of the excised lesions was 14.3 ± 6.7 mm (range: 7–26 mm), and the mean volume of the lesions was 1.6 ± 1.6 mL (range: 0.1–4.8 mL). The mean number of cylinders removed was 13.6 ± 7.1 (range: 6–25).

**Figure 3:**
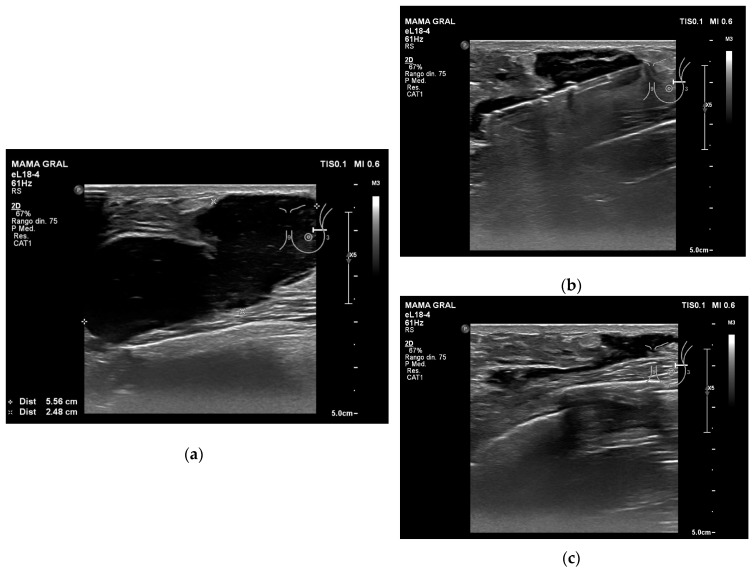
Example of VAB evacuation of a haematoma after VAB excision of a fibroadenoma: (**a**) the ultrasound image shows a hypoechoic-heterogeneous haematoma; (**b**) a vacuum-assisted biopsy needle was inserted into the haematoma cavity and we evacuated fluid until the collection disappeared; (**c**) residual fluid.

The US appearance of the 8 haematomas in this study was a mixture of hyperechoic-heterogeneous (*n =* 4) and hypoechoic-heterogeneous (*n =* 4). The mean time between VAB excision of the lesion and VAB evacuation of the haematoma was 10.1 ± 3.9 days (range: 10–14 days). The average duration of the VAB evacuation of the haematoma was 5.8 ± 1.3 min (range: 4–8 min). Only one patient had VAB evacuation of the haematoma 24 h after VAB excision of the lesion due to intense pain (VAS = 7), which managed to reduce the pain (VAS = 5). In this case, we also observed a reduction in the maximum diameter and volume of the haematoma: from 59 mm to 22 mm and from 27.8 mL to 22 mL, respectively. Table 2 shows the parameters before and after VAB evacuation of the haematomas. The mean intensity of pain before the technique was 5.6 ± 0.7 (range: 5–7), and at 24 h, 1.2 ± 0.7 (range: 0–2). The mean maximum diameter and volume of the haematomas before and after the procedure were also reduced: from 39.1 ± 13.9 mm (range: 23–59 mm) to 17.8 ± 6.8 mm (range: 10–30 mm), and from 20.8 ± 13.5 mL (range: 4.2–44.5 mL) to 2.2 ± 1.1 mL (range: 1.4–4.8 mL), respectively. All the cases in the present study showed success with the VAB system (Figure 3), and the patients tolerated the procedure very well.

Applying Spearman’s *r* correlation coefficient, we observed a very positive correlation between the maximum diameter of the lesion and the maximum diameter and volume of the haematomas 24 h after the procedure, with *r =* 0.87 and *r =* 0.79, respectively. We also correlated the maximum diameter and volume of the excised lesions with the size of the residual haematoma after VAB drainage, which gave *r =* 0.89 for diameter and *r* = 0.74 for volume. All the nonsymptomatic haematomas in this study were absorbed completely within 6 months’ follow-up, and no complications were observed.

## 4. Discussion

To our knowledge, this is the first study in which symptomatic breast haematomas have been evacuated using the VAB system. The results obtained show that the procedure was successful in all cases as it relieved the patients’ pain and reduced the volume of their haematomas. VAB has become recognised in recent years as a safe, cost-effective alternative to open surgery for the removal of benign or probably benign breast lesions [3,4,5,6,7,8]. Haematoma is the most frequent complication after VAB excision of a lesion, with occurrence ranging from 8% to 27% [7,8,10]. Traditionally, the treatment of breast haematoma is conservative in most patients. However, percutaneous drainage with a catheter is used in cases of symptomatic haematomas or large haematomas, although this procedure usually fails because the haematoma is organised with a hyperechoic-hetergeneous US appearance. Complete evacuation of the haematoma was not achieved in one of our cases because it was performed 24 h after VAB excision of the lesion due to the intense pain reported by the patient. In this case, a second VAB evacuation was done 14 days later, which did prove successful. It is worth noting that evacuation was achieved in all the haematomas in the present study when performed 10–14 days after VAB excision of the lesion. The US appearance of the haematomas is fundamental for selecting the day to apply VAB evacuation: a hypoechoic appearance, even if it is partial, indicates the degree of liquefaction of the haematoma and a greater ease for evacuation; however, a hyperechoic appearance is associated with an organised haematoma with clots, which usually makes evacuation difficult. In these cases, the VAB procedure using the aspiration and biopsy options is an efficient technique that achieves a recurrence-free resolution of the haematomas. Nevertheless, determining the ideal day to apply VAB evacuation of the haematomas is fundamental, as it could be done earlier, e.g., between the 8th and 10th day post-excision.

The size of the breast lesion excised with the VAB system is one of the factors related to haematoma formation after the procedure. Studies have been published claiming that the size of the excised lesion does not influence subsequent haematoma formation [5,8,11]. However, our study shows a significant correlation between the size of the excised lesions and the formation of haematomas. One recent study [12] also observed that an increase in lesion size and volume was associated with a higher risk of clinically significant haematoma. We consider it very important to prevent haematomas after VAB excision by applying compression to the bed of the cavity of the excised lesion and advising patients of the care they should take over the following days in order to prevent traumatisms or an increase in tension in the area of interest.

Obvious limitations of our study are the retrospective nature and the relatively small number of patients. However, the data from our study do demonstrate the clinical safety and efficacy of VAB for breast haematomas following excision of the lesion. We consider that a control group would not be necessary because it is unethical to subject patients to intense pain, a multitude of painkillers or surgical treatment in studying a less invasive alternative.

## 5. Conclusions

To our knowledge, this is the first study in which the VAB system has been used to evacuate symptomatic breast haematomas following VAB excision of benign breast lesions. The results obtained show that the technique is safe, efficient and well-tolerated by the patients.

## Figures and Tables

**Table 1:** Characteristics of patients with haematomas.

Patient No.	Age (Years)	Maximum Lesion Diameter (mm)	Lesion Volume (mL)	BI-RADS Category	Number of Cores of Initial BAV	Histopathology
1	37	7	0.12	3	6	Fibroadenoma
2	43	8	0.15	4a	10	Fibroadenoma
3	39	16	1.50	3	15	Fibroadenoma
4	59	12	0.9	4a	14	Fibroadenoma
5	26	22	4.80	3	23	Fibroadenoma
6	36	14	1.10	3	10	Fibroadenoma
7	45	26	3.50	4a	25	Fibroadenoma
8	55	10	1.20	4a	6	Adenosis

**Table 2:** Parameters before and 24 h after VAB evacuation of haematomas.

Patient No.	Before			After		
	Maximum Diameter (mm)	Volume (mL)	VAS	Maximum Diameter (mm)	Volume (mL)	VAS
1	28	4.50	5	10	1.5	1
2	26	4.20	5	12	1.4	0
3	37	19.5	5	20	4.8	2
4	32	15.4	5	14	1.7	1
5	37	30.1	6	18	2.1	2
6	46	44.5	6	25	3.2	1
7	56	29.3	6	30	1.7	1
8	34	19.4	5	14	1.8	1
